# Galectin-3, Inflammation, and the Risk of Atrial High-Rate Episodes in Patients with Dual Chamber Pacemakers

**DOI:** 10.3390/ijms24097710

**Published:** 2023-04-23

**Authors:** Gelu Radu Simu, Raluca Tomoaia, Radu Ovidiu Rosu, Gabriel Gusetu, Mihai Puiu, Gabriel Cismaru, Bogdan Caloian, Andreea Terec, Teodor Buliga, Armand Boer, Ioan Alexandru Minciuna, Gyorgy Bodizs, Dumitru Zdrenghea, Dana Pop

**Affiliations:** 1 5th Department of Internal Medicine, Faculty of Medicine, “Iuliu Hațieganu” University of Medicine and Pharmacy, 400012 Cluj-Napoca, Romania; simugelu@yahoo.com (G.R.S.);; 2 Cardiology Department, Rehabilitation Hospital, 400347 Cluj-Napoca, Romania; 3 Clinical Laboratory, Rehabilitation Hospital, 400347 Cluj-Napoca, Romania

**Keywords:** atrial high-rate episodes, AHREs, galectin-3, inflammation

## Abstract

Atrial high-rate episodes (AHREs) are atrial tachyarrhythmias that are exclusively detected by cardiac implantable electronic devices (CIEDs) with an atrial lead. The objective of this study was to investigate the incidence and predictive factors for AHREs, and to evaluate the ability of inflammation biomarkers to predict the occurrence of AHREs. 102 patients undergoing CIED procedure who received a dual chamber pacemaker were included. CIED interrogation was performed 1 year after the implantation procedure. Patients were divided into groups according to the occurrence of AHREs, which was the primary endpoint of the study. The mean age of the patients was of 73 ± 8.6 years and 48% were male. The incidence of AHREs was 67% at 1 year follow-up. Patients with AHREs were older, had higher left atrial indexed volume (LAVi), higher baseline galectin-3 levels (1007.5 ± 447.3 vs. 790 ± 411.7 pg/mL) and received betablockers more often, along with amiodarone and anticoagulants. Interestingly, the CHADSVASC score did not differ significantly between the two groups. A cut-off value of galectin > 990 pg/mL predicted AHREs with moderate accuracy (AUC of 0.63, 95% CI 0.52 to 0.73, *p* = 0.04), and this association was confirmed in the univariate regression analysis (OR 1.0012, 95% CI 1.0001 to 1.0023, *p* = 0.0328). However, based on the multivariate regression analysis, galectin lost its prognostic significance under the effect of LAVi, which remained the only independent predictor of AHREs (OR 1.0883, 95% CI 1.0351 to 1.1441, *p* = 0.0009). AHREs are common in CIEDs patients. Galectin-3 may bring additional data in the prediction of AHREs.

## 1. Introduction

Atrial high-rate episodes (AHREs) are atrial tachyarrhythmias that are exclusively detected by cardiac-implantable electronic devices (CIEDs) with an atrial lead. For an atrial arrhythmia to be classified as an AHRE, it must fulfil specific criteria: the atrial rate must be ≥175 bpm, and the episode longer than 5 min [1]. Furthermore, AHREs need to be visually inspected by an experienced operator in order to exclude possible false positive results [1].

AHREs are a distinct arrhythmic entity and must be differentiated from clinical atrial fibrillation (AF), which implies a higher arrhythmic burden, a greater impact on morbidity and mortality, and is diagnosed through surface electrocardiographic methods [1,2,3]. This entity also includes atrial tachycardia, atrial flutter and SVT. Moreover, studies have shown that AHREs have different predictors when compared to AF, and are not temporally associated with the risk of stroke [4,5].

Taking into consideration the increasing number of CIEDs, AHREs represent a new arrhythmic entity which is frequently diagnosed. However, the predictive factors and optimal management of AHREs are an ongoing matter of debate.

The objective of this study was to investigate the incidence and predictive factors for AHRE. Furthermore, we evaluated the ability of inflammation biomarkers, including galectin-3 (Gal-3), a novel inflammation biomarker that is also involved in fibrogenesis and atrial remodeling, to predict the occurrence of AHREs.

## 2. Results

### 2.1. Baseline Characteristics

**Figure 1:**
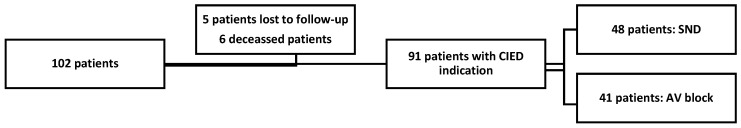
Study flowchart.

Between September 2019 and September 2021, 102 patients (73 ± 8.6 years; 48% male) underwent a dual chamber pacemaker implantation procedure. The rate lost to follow-up was 5%. Six patients died during the follow-up time from causes unrelated to the implantation procedure. Out of the 91 remaining patients, 48 patients (52%) received a pacemaker for sinus node disease and 43 patients (48%) for AV block (Figure 1). In terms of baseline characteristics, patients with a recorded AHRE were older, had a higher left atrial indexed volume, a higher baseline galectin-3 level (790 ± 411.7 pg/mL in the no-AHRE group vs. 1007.5 ± 447.3 pg/mL in the AHRE group) and received more betablockers, amiodarone and anticoagulant therapy more often. Interestingly, the CHADSVASC score did not differ significantly between the two groups.

Detailed patient characteristics can be found in Table 1.

### 2.2. AHRE Incidence and Predictors

**Figure 2:**
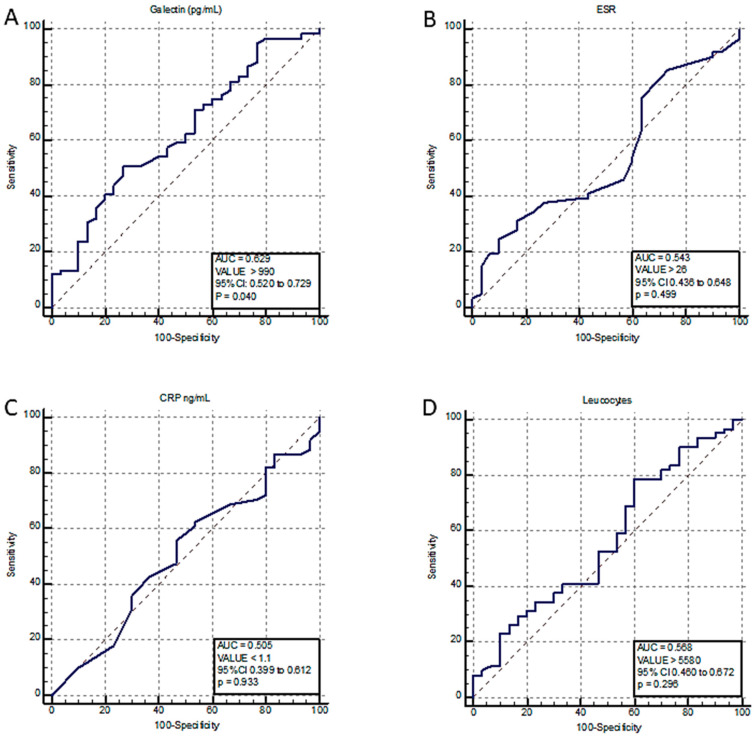
ROC curves used in the prediction of AHREs for (**A**): Galectin-3; (**B**): Erythrocyte sedimentation rate; (**C**): C-reactive protein; (**D**): Leucocytes. AUC, area under the curve; CI, confidence interval; Gal-3, galectin-3; CRP, C-reactive protein; ESR, erythrocyte sedimentation rate; ROC, receiver operating characteristics.

In our study, the AHRE incidence was 67% (*n* = 61) at 1 year follow-up. We aimed to evaluate the impact of galectin-3 on the occurrence of AHREs. Using ROC curve analysis for AHRE identification, we determined a cut-off value of galectin >990 pg/mL to predict AHREs with moderate accuracy (AUC of 0.63, 95% CI 0.52 to 0.73, *p* = 0.04) (Figure 2A). None of the other inflammation parameters were associated with the occurrence of AHREs (Figure 2B–D). 

We sought to determine whether there were any additional factors apart from galectin that might affect the occurrence of AHREs. Thus, in the univariate regression analysis, galectin (OR 1.0012, 95% CI 1.0001 to 1.0023, *p* = 0.0328), age and left atrial indexed volume (LAVi) were associated with the presence of AHREs (Table 2). However, upon multivariate regression analysis, galectin lost its prognostic significance under the effect of LAVi, which remained the only independent predictor of AHREs (OR 1.0883, 95% CI 1.0351 to 1.1441, *p* = 0.0009) (Table 3).

## 3. Discussion

AHREs are a distinct arrhythmic entity and must be differentiated from clinical atrial fibrillation (AF), which entails a higher arrhythmic burden and a greater impact on morbidity and mortality [1,2,3]. CIED-detected AHREs are associated with a two-fold increase in stroke risk when compared to patients with no AHRE, but the risk is significantly lower than the stroke risk of clinical AF [1,2,3]. Moreover, as opposed to AF, AHREs do not seem to be temporally associated with stroke [4,5].

The main findings of our cohort study are: (1) 67% of the CIED recipients have AHRE, (2) age, increased LA indexed volume and galectin-3 were all associated with an increased risk of developing AHRE, (3) a cut-off value of >990 pg/mL was able to predict the presence of AHREs with moderate accuracy.

In previous studies, the reported incidence of AHREs varied between 30 and 70%, depending on the different definitions used to define AHRE [6,7]. In our study, only episodes longer than 5 min were taken into account. The relatively high incidence of AHREs observed in our study might be explained by the more difficult access to rhythmology centers and the high prevalence of cardiovascular risk factors in the Romanian population (sedentary lifestyle, cigarette smoking, arterial hypertension, obesity, metabolic syndrome, and diabetes). AF was present in 67% of the patients who developed AHRE.

Galectin-3 is a member of the β-galactoside-binding lectin family, which binds matrix proteins [8]. Galectin-3 plays a role in the conversion of fibroblasts into myofibroblasts, and enhances the release of proteins involved in fibrogenesis [9]. Galectin-3 is therefore involved in atrial structural and electrical remodeling, and plays a role in AF development and progression [10]. Increased galectin-3 levels have also been shown to play a role in AF recurrence [11]. 

According to Aksan et al., CRT patients who had high galectin-3 levels were more likely to develop AHREs [12]. However, although Gal-3 was not identified as an independent predictor in our study’s multivariate analysis, it was related with a higher risk of developing AHREs in the univariate analysis. These results may be explained by the relatively small sample of patients. We found that a cut-off value for galectin-3 of >990 pg/mL may predict the occurrence of AHREs with moderate accuracy, indicating that this novel marker may be useful in predicting AHREs.

Interestingly, none of the other inflammatory marker, leucocyte, hsCRP and erythrocyte sedimentation rates were associated with AHREs. These results contrast with the results obtained by Pastori et al., where higher levels of CRP and white cell count were associated with AHREs [13]. The observation that galectin-3 was associated with AHREs in our study, but not with the other inflammatory markers, may suggest that Gal-3 is more effective in predicting AHREs. Further studies are needed to certify this finding.

In our study age, an increased LA indexed volume and galectin-3 were all associated with an increased risk of developing AHRE. However, on the multivariate regression analysis, only the LA indexed volume remained an independent predictor of AHREs. Our study obtained similar results with Chen et al. regarding the relationship between increased left atrial size and AHREs. In this study, the left atrial diameter independently predicted AHREs (HR 1.559, 95% CI 1.038–2.341, *p* = 0.033) [7]. Another study showed that even a small increase in LA size could predict AHREs. Kim et al. showed that a LA diameter >41 mm was associated with an increased risk of developing AHREs longer than 6 min [14]. Although we used LAVi, our study obtained similar results. LAVi was the only independent predictor of AHREs in the multivariate regression analysis. Our results suggest that the echocardiographic evaluation of patients before CIED implantation should include measurement of left atrial volume, which may provide early prediction of AHREs.

AF was present in 67% of the patients who developed AHREs, suggesting a common pathogenic mechanism. This finding may support the indication for stroke prevention in patients with AHREs.

### Limitations

This was a single-center study performed in a hospital-based setting. The number of participants was limited. Additionally, not all AHRE electrograms were stored by the device. The device diagnostic information was, however, reviewed by at least one experienced rhythmologist. Different cut-off values of AHRE duration were not established, only episodes longer than 5 min were considered.

## 4. Materials and Methods

### 4.1. Study Population

Patients (102) undergoing a dual chamber pacemaker implantation procedure at the Rehabilitation Hospital in Cluj-Napoca, Romania, between September 2019 and December 2021, were included. All patients received a dual chamber pacemaker. Patients with single chamber pacemakers were excluded. Prior to the procedure, personal medical history, information on co-morbidities and treatment were collected. Blood samples, including high-sensitivity C-reactive protein (hs-CRP) and Gal-3, were obtained, and a transthoracic echocardiography was performed in all patients. The following kits were used to measure galectin-3 and hsCRP levels: EIAab Galectin-3 Elisa kit, EIAab Human PCR Elisa Kit.

The primary endpoint of the study was the occurrence of AHREs, defined as >175 bpm and lasting >5 min. Baseline patient characteristics with and without AHREs were compared. The decision to implant a CIED was based on the recommendations of the 2021 ESC Guidelines on cardiac pacing and cardiac resynchronization therapy [15].

All patients provided written informed consent of the implantation procedure and all pre- and post-implantation diagnostics. The study was carried out according to the principles of the Declaration of Helsinki, and was approved by the local medical ethics committee of the Rehabilitation Hospital of Cluj-Napoca.

### 4.2. Implant Procedure

The procedures were performed by acquiring venous access using the axillary, subclavian or cephalic veins, based on the discretion of the operator. The following cut-off values were considered acceptable for the atrium: sensitivity > 1.5 mv, pacing threshold < 1.0 V@ 0.4 msec, impedance between 300 and 1000 ohm, slew rate > 0.5 V/s; and ventricle: sensitivity > 5 mv, pacing threshold 1.0 V@ 0.4 msec, impedance between 300 and 1000 ohm, slew rate 1.0 V/s. If the optimal values could not be obtained, a new location with the best individual parameters was selected.

All patients received a Medtronic CIED, SEDR01 Sensia DR. All procedures were performed by experienced operators, each having done >500 CIED procedures.

### 4.3. Follow-Up

CIED interrogation was performed at 6 weeks and 1 year after the implantation procedure. Only AHREs longer than 5 min were taken into consideration. Anticoagulants were prescribed following the recommendations of the 2020 Guidelines for the Management of Atrial Fibrillation [1].

### 4.4. Statistical Methods

Normality was tested using the Kolmogorov–Smirnov test. Continuous variables were expressed as the mean ± SD or median (IQR), in agreement with the distribution of the data, whereas categorical variables were presented as percentages. The patients were classified based on the presence or absence of AHREs. *T*-tests and the Mann–Whitney U tests were used to evaluate continuous variables based on their type and distribution, whereas the chi2 test was used to compare categorical data. The prognostic value of each inflammatory biomarker in predicting the development of AHREe was determined using receiver operating curves (ROCs), univariate and multivariate logistic regression analysis. The ROC curves were utilized to establish the optimal cut-off values for different biomarkers for predicting AHREs. The threshold *p*-value to enter the multivariate analysis was <0.3, and the parameters were only regarded as significant in the multivariate analysis if *p* < 0.005. Statistical analysis was conducted using the MedCalc Statistical Software 19.6.1 (MedCalc Software Ltd., Ostend, Belgium; http://www.medcalc.org; 2020, accessed on 25 February 2023). A *p*-value of <0.05 was considered significant.

## 5. Conclusions

AHREs are common in CIED patients. Galectin-3 may bring additional data in the prediction of AHREs.

## Figures and Tables

**Table 1:** Baseline characteristics of patients according to AHRE occurrence.

	AHRE > 5 min No	AHRE > 5 min Yes	*p*-Value
AF any type, *n*, (%)	5 (16.7%)	41 (67.3%)	<0.0001
Age, mean (SD), years	69 ± 8.2	74 ± 8.3	0.0084
Left atrium diameter, mm	39.03	44.95	<0.0001
BMI, mean (SD), kg/m^2^	29.36 ± 5.5	29.02 ± 5.0	0.7747
CRP, mean (SD), mg/mL	2.2 ± 3.3	2.8 ± 4.4	0.5822
Galectin-3, mean (SD), pg/mL	790 ± 411.7	1007.5 ± 447.3	0.0286
Creatinine, mg/dl	0.95 [42.8]	1.09 [47.6]	0.3983
EF, mean (SD), (%)	57.7 ± 8.4	53.7 ± 11.5	0.0964
LAVi, mean (SD), mL/m^2^	31.7 ± 8.4	47.9	<0.0001
ESR, mean (SD), mm/hr	15.2 ± 12.4	19.6 ± 18.9	0.2477
Amiodarone (%)	2.5	45	0.0003
1C antiarrhythmic (%)	5.12	15	0.3579
Anticoagulant therapy (%)	2.5	77.3	<0.0001
Betablocker (%)	38.4	96.2	0.0008
Coronary artery disease (%)	20.5	18.8	0.2501
Dilated cardiomyopathy (%)	2.5	13.2	0.1997
COPD (%)	10.2	13.2	0.7994
Diuretic (%)	10.2	38.9	0.0173
Male (%)	35.8	54.7	0.9377
Arterial hypertension (%)	64.1	98.1	0.8131
HFrEF (%)	12.82	49	0.0.146
ACEi (%)	35.8	62.2	0.5072
Valvular heart disease (%)	61	60.3	0.1378

AHRE, atrial high-rate episodes; AF, atrial fibrillation; BMI, body mass index; EF, ejection fraction; LAVi, left atrial volume indexed to BSA; ESR, erythrocyte sedimentation rate; HFrEF, heart failure with reduced ejection fraction; ACEi, angiotensin-converting-enzyme inhibitors.

**Table 2:** Univariate logistic regression analysis for the occurrence of AHREs. AHRE, atrial high-rate episodes; BMI, body-mass index; EF, ejection fraction; LAVi, left atrial volume indexed to BSA; ESR, erythrocyte sedimentation rate; HFrEF, heart failure with reduced ejection fraction; ACEi, angiotensin-converting-enzyme inhibitors; ARB, angiotensin receptor blocker.

Variable	OR	95% CI	*p*-Value
Galectin-3	1.0012	1.0001 to 1.0023	0.0328
CRP	1.0326	0.9220 to 1.1565	0.5789
ESR	1.0176	0.9878 to 1.0483	0.2508
Leucocytes	1.0001	0.9999 to 1.0004	0.2085
Age	1.0755	1.0150 to 1.1397	0.0137
LAVi	1.1002	1.0484 to 1.1545	0.0001
Gender = “M”	1.4097	0.3340 to 5.9493	0.6402
Arterial hypertension	0.7416	0.1087 to 5.0583	0.7603
BMI	0.9793	0.8640 to 1.1100	0.7437
CHADSVASC score	1.2327	0.8028 to 1.8930	0.3390
Ischemic heart disease	0.9665	0.2258 to 4.1381	0.9634
Amiodarone	2.9726	0.2609 to 33.8725	0.3802
Class 1C antiarrhythmics	1.4505	0.1439 to 14.6192	0.7524
ACEi	0.5604	0.1652 to 1.9004	0.3526
ARB	0.6636	0.2268 to 1.9418	0.4541
Betablocker	0.1593	0.0535 to 0.4743	0.0010
Creatinine	0.9985	0.9882 to 1.0089	0.7761
Dyslipidemia	1.4866	0.5379 to 4.1084	0.4446
E/e′	0.9866	0.8407 to 1.1578	0.8690
Ejection fraction	0.9864	0.9290 to 1.0473	0.6535
Pulmonary hypertension	0.7187	0.1415 to 3.6506	0.6904
HFrEF	2.8392	0.7435 to 10.8429	0.1269
Mitral regurcitation	2.2642	0.8142 to 6.2962	0.1173
Valvular heart disease	1.9740	0.8032 to 4.8517	0.2151

**Table 3:** Multivariate regression analysis for the occurrence of AHREs. LAVi, left atrial indexed volume.

Variable	Coefficient	Std. Error	*p*-Value	OR	95% CI
Galectin-3	0.0016302	0.00098842	0.0991	1.0016	0.9997 to 1.0036
Age	−0.031035	0.058569	0.5962	0.9694	0.8643 to 1.0874
LAVi	0.12206	0.040942	0.0029	1.1298	1.0427 to 1.2242
Betablocker	−0.88367	0.77187	0.2523	0.4133	0.0910 to 1.8761

## Data Availability

The data underlying this article will be shared on reasonable request to the corresponding author.
